# Clustering and meso-level variables in cross-sectional surveys: an example of food aid during the Bosnian crisis

**DOI:** 10.1186/1472-6963-11-S2-S15

**Published:** 2011-12-21

**Authors:** Neil Andersson, Gilles Lamothe

**Affiliations:** 1 Centro de Investigación de Enfermedades Tropicales (CIET), Universidad Autónoma de Guerrero, Calle Pino, El Roble, Acapulco, México; 2 Department of Mathematics, University of Ottawa, Canada

## Abstract

**Background:**

Focus groups, rapid assessment procedures, key informant interviews and institutional reviews of local health services provide valuable insights on health service resources and performance. A long-standing challenge of health planning is to combine this sort of qualitative evidence in a unified analysis with quantitative evidence from household surveys. A particular challenge in this regard is to take account of the neighbourhood or clustering effects, recognising that these can be informative or incidental.

**Methods:**

An example of food aid and food sufficiency from the Bosnian emergency (1995-96) illustrates two Lamothe cluster-adjustments of the Mantel Haenszel (MH) procedure, one assuming a fixed odds ratio and the other allowing for informative clustering by not assuming a fixed odds ratio. We compared these with conventional generalised estimating equations and a generalised linear mixed (GLMM) model, using a Laplace adjustment.

**Results:**

The MH adjustment assuming incidental clustering generated a final model very similar to GEE. The adjustment that does not assume a fixed odds ratio produced a final multivariate model and effect sizes very similar to GLMM.

**Discussion:**

In medium or large data sets with stratified last stage random sampling, the cluster adjusted MH is substantially more conservative than the naïve MH computation. In the example of food aid in the Bosnian crisis, the cluster adjusted MH that does not assume a fixed odds ratio produced similar results to the GLMM, which identified informative clustering.

## Introduction

In public health we often need to understand the change in outcomes associated with a given programme intervention. A household cross-sectional survey might identify the proportion of households covered by an intervention, like food aid. Repeat surveys might detect a change in status, like household food security. The challenge is to work out what the difference in status (improved household food security) has to do with the programme input (management of food aid), particularly in the light of other differences between households that receive food aid and those that do not.

Large scale pragmatic randomised controlled trials can help to sort out causality by demonstrating benefit in sites with the programme compared with those without. In many settings, including evaluation of emergency relief programmes, controlled trials are not an option and working conclusions must be drawn from cross-sectional surveys.

These do not always produce clear evidence, but their relevance to decisions about causal relations is increased when analysis allows exclusion of other explanations (apart from the programme in question) for differences between two time points or between two subgroups. The analysis takes into account potential co-determinants and confounders at different levels (individual, household, cluster, district, region).

There are good reasons for considering potentially causal factors from higher levels of sample aggregation above individual or household in cross sectional studies – like whole cluster or group of clusters. One reason is economy of data collection, avoiding unnecessary repetition of household questions. Information obtained directly from the service provider can be in informative contrast to household data, for example in relation to official fees. Some information, like clinic opening time, is the same for everyone in a given coverage area, so there is little point asking every single household about when clinics are open. The shared data on opening times across communities can be considered “meso-data” – data pertaining to the levels between “micro” (individual or household) and “macro” (for example, national).

Provided the survey to obtain data on higher levels of aggregation includes the same domains (cluster, region, or group of clusters with shared characteristics) and is coterminous with the domains for quantitative study, it is possible to use this characteristic as describing an aspect of the domain. The characteristic can be qualitative or quantitative. The term “meso-analysis” arose in the 1990s with the use of the MH procedure to link coterminous (boundaries end at same place) quantitative and qualitative measurement [1,2]. Meso-level data can also reflect the programme environment or service availability that conditions individual or household health outcomes. The environment includes customs that condition individual outcomes, the “way of doing things” that is linked with health choices.

Key informant interviews are one way to collect meso-level data. Service workers (health, education or other sectors), traditional healers, religious leaders, teachers and shopkeepers are often sources of information. In the aftermath of a devastating measles epidemic in the Mexican state of Guerrero, key informants provided prices of funerals and details of the vaccination campaign for an analysis of costs to the community and services based on data health centres in sentinel sites. This allowed understanding of site specific coping strategies [3]. Coming from the same domain as a household cluster survey, data linkages are straightforward. The problem is how to handle them in the analysis. In particular, one needs to respect if the clustering is part of the causal chain, or if it is simply a nuisance resulting in overestimated statistical confidence.

## Approaches to analysis of correlated cluster data

Pivotal to analysing clustered data is the understanding that clustering can be informative or incidental. In the case of insufficient food aid, informative clustering might be the shared experience of cluster residents when their food distribution agency diverts food supplies for other purposes. An example of incidental clustering might be an association between male absent households and food insufficiency. There may well be an association between these two, but the association is not dependent on place.

A body of literature explores challenges of clustered analysis across different levels of a multi-stage sample. Options include generalised estimating equations, multilevel analysis, also known as random effects logistic regression, and various cluster adjustments of the Mantel Haenszel procedure.

Generalised estimating equations (GEE) have been around for more than two decades [4-6]. The approach is not intended for estimating cluster-level effects on an individual level outcome: for example, the effect of the local programme environment on individual household food security. GEE does not explicitly model between-cluster variation, but focuses on and estimates its counterpart, the within-cluster similarity of the residuals. It uses this estimated correlation to re-estimate the regression parameters and to calculate standard errors. Missing data is a problem with the approach, requiring that missing data records be eliminated prior to computation.

Multilevel analysis attempts to distinguish between informative and incidental clustering [7-9] – sometimes called compositional and textual explanations [10]. MLA explicitly models and estimates the between-cluster variation and incorporates this and the residual variance into standard errors. Within the MLA approaches, the generalised linear mixed model (GLMM) has the advantage of producing estimates of both random effects and fixed effects (hence the term mixed model in GLMM) and it is not incapacitated by missing data. Theoretical drawbacks include reliance on linear models when at least some of the effects at different levels may not be linear [11,12]. There may also be different confounding at different levels. The idea that linked “sub-studies” can increase understanding of confounders [13] is not often a feasible option.

One of the most frequently referenced procedures in the history of statistics, the Mantel Haenszel (MH) computation of fixed effects separates data into strata and, providing there is not excessive heterogeneity between strata, averages the measured effect [14,15]. MH has the important attribute that it is non-parametric, relating the *a* cell in a 2x2 table to the margins, without dependence on the other internal cells. Birch showed that under the assumption that the within table odds ratios are homogeneous the MH test is the uniformly most powerful unbiased test. Furthermore the MH procedure is robust against departures of this assumption of homogeneous association [16]. MH can detect different confounding at different levels; one can stratify the association with a community level variable (a certain type of community) just as one can stratify by a household level variable (like male absent household). Analogous to fixed effect logistic regression and producing close to identical results in large data sets [17], the naïve (non-cluster adjusted) MH largely “neutralises” the fact that data come from different levels, treating meso-variables in the same way as an individual level variable. Compared with MLA and GEE, the MH procedure is simple to compute, it is fairly intuitive and it does not require any assumptions for binomial data. But as with fixed effects logistic regression, the naïve Mantel Haenszel ignores clustering in estimating standard error, leading often to overstatement of statistical confidence (confidence intervals misleadingly narrow).

The large number of proposals to adjust MH for clustering suggests lack of resolution of the issue [18,19]. For example, the statistic of Zhang and Boos [20] adjusts harshly for the dependency between observations of subjects from the same cluster, increasing the confidence intervals roughly proportionally to the intra-cluster correlation coefficient. Furthermore the Zhang and Boos procedure assumes that the exposure and covariate are cluster specific. This assumption is too restrictive for many studies. Like the naïve MH approach, this cluster adjustment does not distinguish between informative and incidental clustering.

## The case of food security in Bosnia

During the Bosnian conflict, a series of surveys collected household data through household interviews. Theoretically all households had received the same amount of free food aid in an international relief effort including universal food distribution. In addition to the households interviewed (contiguous households without sub-sampling in each of 66 clusters), research teams interviewed international relief workers, reviewed food distribution practices and discussed key issues in gender-stratified focus groups. The evaluation had the explicit objective of identifying under-served groups and making policy recommendations to improve equitable delivery of food.

An outcome of interest was food under-supply: those who received food aid but who still reported insufficient food in the last week. Household variables included male absent households, presence of displaced people, ethnicity, employment, disability in the household and crowding (five or more members). Meso-variables included urban/rural (defined by size of community and its characteristics), recent conflict in or near the community; which of five main food aid agencies was responsible for the food delivery (UNHCR, Merhamed, Caritas, Red Cross or the Local Logistics Centre run by the municipality).

For this paper, we reanalysed the factors associated with household food under-supply using five multivariate approaches: (i) the naïve stratified MH; (ii) the Lamothe cluster-adjusted MH which applies a robust variance estimator for cluster-correlated data [21,22], (see Statistical Annex, Additional file 1) to address clustering in a stratified last stage random sample; (iii) generalised estimating equation (GEE), accessed in the R package Zelig [23], applying an exchangeable correlation structure (logit.gee model, 1000 simulations); (iv) the Lamothe cluster adjusted confidence interval that does not assume a fixed effect across clusters, estimating the OR as the midpoint of the confidence interval, and (v) mixed effects modelling using the R package lme4 [24], achieving a fit of fixed and random effects by the Laplace approximation [25].

In each of the five approaches we developed two multivariate models of the effect on the outcome, one of household factors and the other of meso variables. Each initial model began with all candidate variables (above), stepping down one variable at a time using backwards elimination until only statistically significant variables remained in the final model. We then combined the household and meso-variable models and repeated the process to arrive at a final combined model. In the GLMM, we analysed “Republic” (Republika Srpska in contrast with Bosnia and Herzegovina), “Frontline” (denoting active conflict in the month of the survey) and “Rural” (using the standard regional definition of urban/rural) as random effects.

## Results

Table 1 shows the unadjusted Odds Ratios and results of a naïve Mantel-Haenszel stratified analysis, the cluster adjusted Mantel Haenszel (MH), GEE, the Lamothe adjusted OR and GLMM.

**Table 1:** Household risk of food under-supply in Bosnia (still short of food after receiving food aid) from multivariate analysis, 1995 and 1996

Variable	Bivariate Unadjusted OR Cornfield (95%CI)	Naïve Mantel Haenszel OR-adjusted (95%CI)	Lamothe cluster adjusted ORmh (assuming OR constant)§ (95%CIca)	GEE Exchangeable matrix OR (naïve 95%CI)	Lamothe cluster adjusted ORmh (**not** assuming OR constant ¤) (95%CIca)	GLMM Laplace approximation OR (95%CI)
Household characteristics						
Disabled in household	2.24 (1.42-3.25)	1.52 (1.02-2.27)	ns	ns	ns	ns
Displaced people	2.38 (2.18-2.60)	2.36 (2.16-2.58)	2.29 (1.53-3.43)	1.95 (1.56-2.44)	2.43 (1.78-3.08)	2.41 (2.2-2.64)
No remittance	1.84 (1.63-2.10)	1.90 (1.67-2.14)	1.60 (1.28-1.99)	1.68 (1.33-2.09)	1.87 (1.40-2.34)	1.89 (1.67-2.14)
Female headed household	1.12 (0.97-1.28)	1.25 (1.08-1.43)	1.21 (1.01-1.44)	1.27 (1.08-1.49)	1.25 (1.03-1.48)	1.22 (1.06-1.4)
No employment	1.82 (1.65-2.01)	ns	ns	ns	ns	ns
Muslim vs. non-Muslim	0.96 (0.88-1.05)	ns	ns	ns	ns	ns
Cluster characteristics						
Agency2 (vs others)	1.71 (1.55-1.89)	1.65 (1.49-1.82)	ns	ns	1.8 (1.10-2.50)	1.59 (1.43-1.76)
Republic (vs BiH)	1.21 (1.10-1.32)	1.37 (1.24-1.50)	ns	ns	ns	ns
Recent frontline conflict	0.99 (0.89-1.10)	ns	ns	ns	ns	ns
Rural (vs urban)	1.00 (0.88-1.13)	ns	ns	ns	ns	ns
						
Number of households	n=17905	n=17549	n=17562	n=	n=17562	n=17561
						

ns=not significant, dropped from model

¤ odds ratio estimated as the midpoint of cluster-adjusted MH 95%CI

§ data for this calculation provided in Table 2

**Table 2:** Proportions of households *under-supplied* by the food aid programme in groups with different combinations of risk factors: from final model of cluster adjusted Mantel Haenszel analysis, 1995 and 1996

	Proportion	%
Remittance, no DP, other agency	118/1726	6.8
No remittance, no DP, other agency	639/6105	10.5
Remittance, no DP, Agency2	52/477	10.9
Remittance, DP, other agency	67/532	12.5
No remittance, no DP, Agency2	308/1837	16.8
No remittance, DP, Agency2	241/816	19.5
No remittance, DP, other agency	500/2294	21.8
Remittance, DP, Agency2	52/183	28.4

The final models of cluster-adjusted MH and GEE excluded all of the meso-variables whereas the Lamothe adjusted OR and GLMM retained Agency2, the variable identifying the clusters that received food aid from a particular distribution source. The Lamothe adjusted OR produced very similar results to the GLMM in this example – in relation both to the variables retained in the final model and to the size of effect.

Table 2 illustrates the transparency available with stratification used in the MH procedures, allowing detailed review of under-supply across the different risk subgroups. The worst off subgroup were four times more likely to be under-supplied than residents with remittance who were not supplied by Agency2. This also offers some understanding of the meaning of different combinations, or the relevance of each factor in combination with others.

## Discussion

Cluster surveys can produce a mix of qualitative and quantitative variables for each cluster from observation, key informants or focus groups and household questionnaires. The cluster survey approach has many advantages and well known problems. Confounding can still occur at other levels of aggregation not taken into account, and variables that are not measured in the study can cause confounding. A cluster sample will almost invariably have a smaller variance than the same number of households in a simple random sample. This stems from the common sense principle that people who live next door to each other tend to be more similar than those living some households away or in a different town. The resulting concern is that a cluster sample will overstate the statistical confidence in any particular association. The solution of most cluster adjustments is to increase the confidence interval.

This adjustment assumes, however, that the clustering is incidental to the association. Crucially, if the clustering is informative – the factor “works” through its clustered occurrence (such as characteristics of the agency supplying food aid to that cluster) – we would be losing information by simply adjusting confidence downwards without reassessing the risk estimate.

In Table 1, the variables “Disabled in household” and “male absent households” reflect associations with food sufficiency that happen more or less randomly across clusters. There is no *a priori* reason to believe that food sufficiency of disabled people or male absent households might be affected by the particular cluster they live in. Although they were strong factors in a naïve MH analysis, the associations of disabled and male-absence with under-supply disappear with the cluster adjusted MH, GEE, Lamothe adjusted OR and GLMM. The meso-variables Republic (Republic Srpska contrasting Bosnia and Herzegovina) and Rural (contrasting with urban) represent different levels of aggregation above cluster. Unimpressive in the naïve MH, these fall out of all cluster adjusted analysis.

The household variable No Remittance describes households that had not received remitted income from abroad in the past year. Although the proportions receiving these remittances varied from cluster to cluster, it is not cluster *per se* that determined whether individual households received the money. More to the point, cluster did not affect the relationship between remitted income and food sufficiency.

“Agency 2” identifies clusters receiving United Nations food aid through an ethnically based organisation with strong links to the corresponding military. There was knowledge, if not willing acceptance, that this and other ethnically based agencies channelled food to their respective military. Thus, since food quotas were based on known civilian populations, in the distribution sites of the ethnically based distribution agencies there was effectively less food available for civilians. Hence, more households reported undersupply.

The Lamothe adjusted non-fixed OR and GLMM echo the findings of the naïve (not cluster-adjusted) MH with respect to Agency 2. Households supplied by Agency2 received significantly less: an average of 5.22kg per person per month, compared with 6.21kg per person per month (Kruskal Wallis H 167.2, 1df, p=0.000001). The mode of food aid distributed per household by Agency2 was 10kg, compared with 20kg distributed by other agencies.

Both cluster adjusted MH and GEE presume that all clustering is incidental – the former by assuming a fixed OR across clusters and the latter by largely ignoring differences between clusters. Both Lamothe adjusted non-fixed OR and GLMM allow for informative clustering, the former by not assuming a fixed OR across clusters and the latter by allowing separate regression equations across different groups of clusters. While the two methods produced much the same results in this example, both identifying informative clustering, the Lamothe adjusted OR has the advantage of not assuming any particular distribution of the data.

The Bosnian data set has noteworthy characteristics that could influence or even set the conditions for the useful performance of the Lamothe OR in this setting. First, it is of moderately large size (some 17,500 households in the two years studied here) with a large number of sites (around 120 clusters over the two years) and a large number of households in each cluster (average 100). Second, two years of intensive war before the survey might have flattened out differences across the affected area, resulting in only moderate heterogeneity between clusters. Under conditions of greater heterogeneity, one might have to apply the approach to relatively homogeneous subsets.

The apparently useful performance of the Lamothe adjusted OR in this particular case does not detract from the fundamental truth that a cross-sectional study remains a cross sectional study. GEE, GLMM and the Lamothe adjustments do not get around the issues of temporality that limit causal interpretation for observational data. In her comprehensive review of area effects on health, Diez Roux warns against simplistic explanations that reduce area or neighbourhood to “just another variable”. Part of the solution is to get closer to the specific content of the meso-variable, for example, the characteristics of agencies supplying food aid, or the likely clustering of displaced people, whose need for food aid might be greater. To arrive at a working notion of causality, and this is something that one had to attempt with even the flawed data available from the cross sectional studies available in Bosnia, it is the specific character of the meso-variable that matters.

## Conclusion

Shared characteristics at different levels of aggregation can add meaning to cross sectional studies where causal inference is a concern. But with cluster samples come other questions, including whether the clustering is part of the causal chain, or whether it is a nuisance resulting in overestimated statistical confidence.

GEE deals with clustering by modelling the in-cluster association and ignoring the between- cluster variation. GLMM generates separate estimates for an individual predictor and its group-level mean [26], allowing separation of random effects from fixed effects. Under certain conditions and without assumptions about the distribution of the data, the Lamothe MH statistic with non-fixed OR adjusts for clustering and may discriminate usefully between informative and non-informative clustering.

## Competing interests

The authors declare that they have no competing interests.

## Supplementary Material

Additional file 1 Click here for file
