# Supplementary material for: Clustering and meso-level variables in cross-sectional surveys: an example of food aid during the Bosnian crisis
Source: BMC Health Serv Res. 2011 Dec 21;11(Suppl 2):S15. doi: 10.1186/1472-6963-11-S2-S15 (PMC3332559; doi:10.1186/1472-6963-11-S2-S15)
Supplement: Additional file 1 [file 1472-6963-11-S2-S15-S1.pdf]

Adjusting the Mantel Haenszel Test Statistic and Odds Ratio for Cluster Sampling

Gilles Lamothe
Department of Mathematics and Statistics, University of Ottawa

October 15, 2011

1 Introduction

The purpose of this note is to introduce practicing epidemiologist to a robust cluster adjustment of the Mantel Haenszel Test Statistic and Odds Ratio. We will discuss the applicability of the adjustment and also its computation. Throughout we will use data from the file SDZ1.rec which comes with the CIETmap analysis software. We start by giving a short introduction to odds ratio estimation and introduce the cluster adjustment within this simple context. The justification for the method will follow in a biostatics paper. It is an application of the well-known delta method.

2 Odds Ratio and Cluster Adjustment

An odds ratio is a statistic that measures the degree of association between two dichotomous variables, see [3]. Epidemiologist use it as a measure of risk. We compute it as follows OR = $a\,d/(b\,c)$, where referring to the following contingency table.

	Exposure		
Outcome	+	−	Total
+	a	b	$m_1 = a + b$
−	c	d	$m_0 = c + d$
Total	$n_1 = a + c$	$n_0 = b + d$	$T = a + b + c + d$

The odds that an event will occur is the probability that it will occur divided by the probability that it will not occur. With an odds greater than one, the event is more likely to occur than not. An odds ratio is the comparison of the odds for two groups.

As an example we consider the two variables DIARR and CLWATER which are found in the data file SDZ1.rec which comes with the CIETmap analysis software.

	exposure		
outcome	CLWATER =1	CLWATER =2	total
DIARR =1	a=305	b=827	$m_1 = 1132$
DIARR =2	c= 225	d=986	$m_0 = 1211$
total	$n_1 = 530$	$n_0 = 1813$	$T = 2343$

The crude odds ratio is $\text{OR} = a\,d/(b\,c) = 1.62$. We interpret the OR as follows. The odds of a positive outcome is 1.62 larger for someone with a positive exposure compared to someone with a negative exposure.

It is always important to account for sample to sample variability. We can construct a confidence interval to this end. A simple way to construct a confidence interval for the odds ratio is to use a procedure based on Woolf's variance estimator of the log-OR, see [6]. The log odds ratio is $\ln(\text{OR}) = 0.48006$. The variance estimate of the log-OR is

$$\hat{V}[\ln(\text{OR})] = \frac{1}{a} + \frac{1}{b} + \frac{1}{c} + \frac{1}{d} = 0.00995.$$

We start by constructing a 95% confidence interval for the log-OR, which is

$$\ln(\text{OR}) \pm 1.96\sqrt{\hat{V}[\ln(\text{OR})]} = [0.278, 0.669].$$

By exponentiating the lower and upper limit of the confidence interval for the log-OR, we obtain a confidence interval for the odds ratio. A 95% confidence interval for the odds ratio is

$$\exp\left\{\ln(\text{OR}) \pm 1.96\sqrt{\hat{V}[\ln(\text{OR})]}\right\} = [1.32, 1.95].$$

The crude odds ratio is indeed a crude measure of the association in the sense that it does not control for possible confounding. In the case of observational studies, where we do not control (i.e. assign) the exposure, it is possible that the observed association can actually be explained by another factor. It is important to control for such confounding.

Mantel-Haenszel in [2] discuss methods to control for confounding in the case where the confounding variables are categorical. It is a stratified analysis based on the levels of the confounder or effect modifier, called the stratifier. We discuss the cluster adjustment for the Mantel-Haenszel in Section 3.

A stratified analysis such as proposed by Mantel and Haenszel are only valid in the case of a fixed effect factor, that is all levels of the factor are involved in the study. However in some cases, the factor might have a random effect on the outcome and/or the exposure. As an example, many studies are community based and often not all levels the community covariate is involved in the study. This will be the case when a few communities are chosen at random to take part in the study. In this case, a community would be called a cluster.

We allow the clustering to have multiple levels. For example, we could sample districts and then sample towns in those districts and then choose households in those towns. In the latter example the district would be the primary cluster. We will assume that the primary clusters are independent.

We explain the cluster adjustment for the crude odds ratio. The adjustment is similar to the adjustment proposed by [5] in the context of multistage surveys. We propose that is it also valid using a modeling approach. We say that the observations are multinomially clustered, in the sense that we have observations from a sample of clusters and that the observations are then cross classified according to the outcome and the exposure.

The cluster adjustment is quite simple. The first step is to assign a linearized value to each of the T observations which depends on its cross classification. The linearized values are computed as follows.

	Exposure	
Outcome	+	−
+	$d/(a\,d)$	$-c/(b\,c)$
−	$-b/(b\,c)$	$a/(a\,d)$

In our example, the linearized values are

Outcome	Exposure +	Exposure −
+	$d/(a\,d) = 0.003279$	$-c/(b\,c) = -0.001209$
−	$-b/(b\,c) = -0.004444$	$a/(a\,d) = 0.001014$

In our example, the variables SITE is considered as the cluster variable. The variable SITE has 20 levels. For each level of the cluster variable, we will compute the sum of the linearized values and obtain $C = 20$ sums. These sums are

-0.0019	0.0338	-0.0374	0.0733	0.0211
-0.0265	-0.0035	0.0088	-0.0079	-0.0467
-0.0239	-0.013	-0.0396	-0.0148	0.0301
0.0084	-0.0256	0.042	0.0102	0.013

Consider these sums as a random sample of $C = 20$ observations: z_i, for $i = 1, \ldots, C$. Compute the corresponding between cluster variance estimator for a sum (see [4]):

$$\hat{V}_C = \frac{C}{C-1} \sum_{i=1}^{C} (z_i - \overline{z})^2 = 0.01851.$$

where $\overline{z} = \sum_{i=1}^{C} z_i / C$ is the sample mean.

The between cluster variance estimate $\hat{V}_C$ of the total linearized values is the estimated variance for the log-OR. With the estimated variance of the log-OR, we can construct a 95% confidence interval for the log-OR. In our case, the cluster adjusted 95% confidence interval for the log-OR is

$$\ln(\text{OR}) \pm 1.96\sqrt{\hat{V}_C} = [0.207, 0.740].$$

Exponentiating the latter interval gives a 95% confidence interval (cluster adjusted) for the crude odds ratio:

$$\exp\left\{\ln(\text{OR}) \pm 1.96\sqrt{\hat{V}_C}\right\} = [1.23, 2.10].$$

Note that the adjusted interval is 1.37 times the size of the unadjusted interval. A way to measure the cluster effect (sometimes called the design effect) is to compute the ratio of the cluster adjusted variance to the unadjusted variance. The cluster effect in our example is

$$\text{cluster effect } = \frac{\hat{V}_C[\ln(\text{OR})]}{\hat{V}[\ln(\text{OR})]} = 1.86.$$

The cluster effect is a measure of the effective sample size. This means that we need about 1.86 more observations to maintain the same level of precision compared to a sample with no cluster effects.

3 Mantel-Haenszel Methodology

Mantel and Haenszel in [2] proposed stratification techniques to account for confounding. Their methods assume that the confounder has fixed effects. We adjust these methods to also account for a covariate with random effects. In other words, we adjust for clustering.

As in the previous section we consider the variables DIARR and CLWATER as the outcome and exposure variables. The stratifier is the variable HHSIZE which has $S = 3$ levels. For each level s of the stratifier we obtain a contingency table.

	Exposure		
Outcome	+	−	Total
+	a_s	b_s	$m_{1s} = a_s + b_s$
−	c_s	d_s	$m_{0s} = c_s + d_s$
Total	$n_{1s} = a_s + c_s$	$n_{0s} = b_s + d_s$	$T_s = a_s + b_s + c_s + d_s$

Here are the 3 tables.

	Exposure	
Outcome	+	−
+	$a_1 = 100$	$b_1 = 256$
−	$c_1 = 82$	$d_1 = 406$

	Exposure	
Outcome	+	−
+	$a_2 = 92$	$b_2 = 277$
−	$c_2 = 78$	$d_2 = 294$

	Exposure	
Outcome	+	−
+	$a_3 = 113$	$b_3 = 294$
−	$c_3 = 65$	$d_3 = 286$

The Mantel-Haenszel test statistic to test for conditional independence between the outcome and exposure variables is of the form $\chi^2_{MH} = A^2/\mathrm{Var}(A)$, where

$$A = \sum_{s=1}^{S} \frac{a_s d_s - b_s c_s}{T_s} = 48.001.$$

Its unadjusted variance is

$$\mathsf{Var}(A) = \sum_{s=1}^{S} (m_{0s} m_{1s} n_{0s} n_{1s}) / [T_s^2 \, (T_s - 1)] = 101.559,$$

and the unadjusted Mantel-Haensel (as called the MH χ^2 summary statistic) test statistic is $\chi^2_{MH} = \frac{A^2}{\mathsf{Var}(A)} = 22.69$. At a level of significance of 5%, we can conclude by conditioning on the stratifier that the outcome and exposure are not independent, since $\chi^2_{MH} > 3.84$. Note that 3.84 is an upper quantile of order 5% from a chi-square distribution with 1 degree of freedom.

To measure the size of the association, Mantel and Haensel proposed the use of the Mantel-Haensel odds ratio

$$\widehat{\mathrm{OR}}_{MH} = \frac{\sum_{s=1}^{S} a_s d_s / T_s}{\sum_{s=1}^{S} b_s c_s / T_s} = \frac{\sum_{s=1}^{S} w_s \, \widehat{\mathrm{OR}}_s}{\sum_{s=1}^{S} w_s},$$

where

$$w_s = b_s \, c_s / T_s \quad \text{and} \quad \widehat{\mathrm{OR}}_s = \frac{a_s \, d_s}{b_s \, c_s}.$$

In our example, the Mantel-Haenszel odds ratio is

$$\widehat{\mathrm{OR}}_{MH} = \frac{\sum_{s=1}^{S} a_s d_s / T_s}{\sum_{s=1}^{S} b_s c_s / T_s} = 1.61.$$

It can be seen as a weighted average of the varying strata odds ratios.

A 95% (large sample) confidence for the the Mantel-Haenszel odds ratio using Miettinen's procedure, see [7], that does **not** take the clusters into account is

$$\widehat{\mathrm{OR}}_{MH}^{1\pm 1.96/\sqrt{\chi^2_{MH}}} = [1.32, 1.95],$$

where we used the Mantel-Haenszel chi square statistic

$$\chi^2_{MH} = \frac{\left[\sum_{s=1}^{S}(a_s\, d_s - b_s\, c_s)/T_s\right]^2}{\sum_{s=1}^{S}(m_{0s}m_{1s}n_{0s}n_{1s})/[T_s^2\,(T_s-1)]} = \frac{(48.001)^2}{101.559} = 22.69.$$

This is the unadjusted method that is implemented in CIETmap. It should be known that this procedure assumes a common odds ratio of one for all strata since it is based on the Mantel-Haenszel summary χ^2 statistic.

We start by adjusting Miettinen's procedure for clustering and then give an adjustment for the interval of the MH OR that does not assume a common odds ratio. In other words we propose a procedure that is valid even under heterogeneity of association.

3.1 Adjusting Miettinen's Interval

A statistic used in the Mantel-Hanszel summary chi-square test statistic is

$$A = \sum_{s=1}^{S} \frac{a_s d_s - b_s c_s}{T_s} = 48.001.$$

Its unadjusted variance is

$$\mathsf{Var}(A) = \sum_{s=1}^{S}(m_{0s}m_{1s}n_{0s}n_{1s})/[T_s^2\,(T_s-1)] = 101.559,$$

and the unadjusted Mantel-Haensel test statistic is

$$\chi^2_{MH} = \frac{A^2}{\mathsf{Var}(A)} = 22.69.$$

We will compute the variance for A taking into account the clusters. The adjusted variance will be denoted $\mathsf{Var}_{\text{adj}}(A)$. With this adjusted variance, we obtain the adjusted Mantel-Haensel summary chi-square test statistic:

$$\chi^2_{MH}(\text{adj}) = \frac{A^2}{\mathsf{Var}_{\text{adj}}(A)}.$$

The adjusted confidence interval will be of the form:

$$\hat{\Psi}_{MH}^{1\pm z/\sqrt{\chi^2_{MH}(\text{adj})}}.$$

Steps to compute $\mathsf{Var}_{\textbf{adj}}(A)$:

1. Compute the statistic of interest:

$$A = \sum_{s=1}^{S} \frac{a_s d_s - b_s c_s}{T_s} = 48.001.$$

Note: The terms in the above sum will be denoted as follows:

$$A_s = \frac{a_s d_s - b_s c_s}{T_s}.$$

We have $A_1 = 23$, $A_2 = 7$, and $A_3 = 17$.

2. We will assign the linearized value z_{ijs} to the jth unit within the ith cluster within the level s of the stratifier. We determine the linearized value as follows:

If the member has positive exposure and positive outcome, then

$$z_{ijs} = \frac{d_s - A_s}{T_s}.$$

If the member has negative exposure and positive outcome, then

$$z_{ijs} = \frac{-c_s - A_s}{T_s}.$$

If the member has positive exposure and negative outcome, then

$$z_{ijs} = \frac{-b_s - A_s}{T_s}.$$

If the member has negative exposure and negative outcome, then

$$z_{ijs} = \frac{a_s - A_s}{T_s}.$$

In our example, for each level s of the stratifier, the linearized values can take on four different values. For $s = 1$, they are

	exposure	
outcome	CLWATER =1	CLWATER =2
DIARR =1	0.4535	-0.1247
DIARR =2	-0.3308	0.0910

For $s = 2$, they are

	exposure	
outcome	CLWATER =1	CLWATER =2
DIARR =1	0.3869	-0.1152
DIARR =2	-0.3837	0.1142

For $s = 3$, they are

	exposure	
outcome	CLWATER =1	CLWATER =2
DIARR =1	0.3543	-0.1087
DIARR =2	-0.4109	0.1261

We are using the SITE variable to identify a cluster. There are $C = 20$ clusters. There are $T = 2343$ linearized values. One for each ultimate sampling unit. We will compute the sum of the linearized values for each cluster. These $C = 20$ sums are

0.6909	4.6783	-0.2295	10.3969	5.3066
-1.3010	3.1393	3.6256	2.6032	-1.1987
-0.5078	0.4695	-0.0872	0.2459	4.4527
4.0940	0.7732	5.4074	2.4693	2.9727

3. We will compute the between cluster variance estimate, that is we compute the sample variance of the $C = 20$ cluster sums.

$$v_C = \frac{C}{C-1}\left[\sum_{i=1}^{C}(z_{i\cdot\cdot} - \overline{z})^2\right] = 166.609.$$

where $z_{i\cdot\cdot} = \sum_{s=1}^{S}\sum_{j=1}^{T_i} z_{ijs}$ and $\overline{z} = \sum_{i=1}^{C} z_{i\cdot\cdot}/C$.

The estimated variance of A which takes the clustering into account is $\mathsf{Var}_{\text{adj}}(A) = v_C$.

We are now ready to construct a 95% confidence interval for log of the Mantel-Haenzsel OR (taking account of the clustering):

$$\widehat{\text{OR}}_{MH}{}^{1\pm z/\sqrt{\chi^2_{MH}(\text{adj})}} = [1.25, 2.06],$$

where the adjusted chi-square is

$$\chi^2_{MH}(\text{adj}) = \frac{A^2}{\mathsf{Var}_{\text{adj}}(A)} = \frac{(48.001)^2}{166.6088762} = 13.83.$$

We should note that the adjusted interval is 1.29 times larger than the unadjusted interval.

3.2 Robust Cluster Adjustment

We will now describe an alternative cluster adjustment that does not assume a common odds ratio. It is based on the Mantel-Haenszel odds ratio itself, instead of the Mantel-Haenszel chi-square test statistic.

We will start by computing the Mantel-Haenszel log-odds ratio:

$$\ln(\widehat{\text{OR}}_{MH}) = 0.4736.$$

We will compute the variance for $\ln(\widehat{\text{OR}}_{MH})$ taking into account the clusters. The adjusted variance will be denoted $\mathsf{Var}_{\text{adj}}(\ln(\widehat{\text{OR}}_{MH}))$. The adjusted 95% confidence interval for the Mantel-Haenszel odds ratio will be of the form:

$$\exp\{\ln(\widehat{\text{OR}}_{MH}) \pm 1.96\sqrt{\mathsf{Var}_{\text{adj}}(\ln(\widehat{\text{OR}}_{MH})}\}.$$

Steps to compute $\mathsf{Var}_{\textbf{adj}}(\ln(\widehat{\textbf{OR}}_{MH}))$:

1. We will use the following notation:

$$R_s = \frac{a_s\, d_s}{T_s}, S_s = \frac{b_s\, c_s}{T_s}, R = \sum_{s=1}^{S} R_s, S = \sum_{s=1}^{S} S_s.$$

Note that the Mantel-Haenszel odds ratio is of the form:

$$\widehat{\mathrm{OR}}_{MH} = \frac{\sum_{s=1}^{S} a_s d_s / T_s}{\sum_{s=1}^{S} b_s c_s / T_s} = \frac{R}{S} = 1.61.$$

In our example,

$$R = R_1 + R_2 + R_3 = 48.1042654 + 36.50202429 + 42.63588391 = 127.2421736$$

and

$$S = S_1 + S_2 + S_3 = 24.87203791 + 29.15789474 + 25.21108179 = 79.24101445.$$

2. We will assign the linearized value z_{ijs} to the jth unit within the ith cluster within the level s of the stratifier. We determine the linearized value as follows:

If the member has positive exposure and positive outcome, then

$$z_{ijs} = \frac{d_s/R - (R_s/R - S_s/S)}{T_s}.$$

If the member has negative exposure and positive outcome, then

$$z_{ijs} = \frac{-c_s/S - (R_s/R - S_s/S)}{T_s}.$$

If the member has positive exposure and negative outcome, then

$$z_{ijs} = \frac{-b_s/S - (R_s/R - S_s/S)}{T_s}.$$

If the member has negative exposure and negative outcome, then

$$z_{ijs} = \frac{a_s/R - (R_s/R - S_s/S)}{T_s}.$$

In our example, for each level s of the stratifier, the linearized values can take on four different values. For $s = 1$, they are

	exposure	
outcome	CLWATER =1	CLWATER =2
DIARR =1	0.0037	-0.0013
DIARR =2	-0.0039	0.0009

For $s = 2$, they are

outcome	exposure CLWATER =1	CLWATER =2
DIARR =1	0.0032	-0.0012
DIARR =2	-0.0046	0.0011

For $s = 3$, they are

outcome	exposure CLWATER =1	CLWATER =2
DIARR =1	0.0029	-0.0011
DIARR =2	-0.0049	0.0011

We are using the SITE variable to identify a cluster. There are $C = 20$ clusters. There are $T = 2343$ linearized values. One for each ultimate sampling unit. We will compute the sum of the linearized values for each cluster. These $C = 20$ sums are

-0.0034	0.0319	-0.0433	0.075	0.0254
-0.0261	-0.0009	0.0095	-0.0081	-0.0522
-0.022	-0.013	-0.042	-0.0137	0.0304
0.0097	-0.0186	0.0392	0.0111	0.011

3. We will compute the between cluster variance estimate of $\sum z_{ijs}$. The between cluster variance estimate is
$$v_C = \frac{C}{C-1}\left[\sum_{i=1}^{C}(z_{i\cdot\cdot} - \overline{z})^2\right] = 0.0195,$$
where $z_{i\cdot\cdot} = \sum_{s=1}^{S}\sum_{j=1}^{T_i} z_{ijs}$ and $\overline{z} = \sum_{i=1}^{C} z_{i\cdot\cdot}/C$.

The estimated variance of $\ln(\widehat{\mathrm{OR}}_{MH})$ which takes the clustering into account is
$$\mathsf{Var}_{\mathrm{adj}}(\ln(\widehat{\mathrm{OR}}_{MH})) = v_C = 0.0195.$$

We are now ready to construct a 95% confidence interval for the Mantel-Haenszel odds ratio (taking account of the clustering):
$$\exp\left\{\ln(\widehat{\mathrm{OR}}_{MH}) \pm 1.96\sqrt{\mathsf{Var}_{\mathrm{adj}}(\ln(\widehat{\mathrm{OR}}_{MH}))}\right\} = [1.22, 2.11].$$

Note that the adjusted interval is 1.41 times larger than the unadjusted interval. A measure of the cluster effect is the ratio between the cluster adjusted variance against the unadjusted variance. It is
$$\text{cluster effect} = \frac{\mathsf{Var}_{\mathrm{adj}}(\ln(\widehat{\mathrm{OR}}_{MH})}{\mathsf{Var}(\ln(\widehat{\mathrm{OR}}_{MH})} = 1.95.$$

Here we computed the unadjusted variance using the RBG formula from [8], which is
$$\mathsf{Var}(\ln(\widehat{\mathrm{OR}}_{MH}) = \frac{\sum_{s=1}^{S} R_sP_s}{2R^2} + \frac{\sum_{s=1}^{S} P_sS_s}{2RS} + \frac{\sum_{s=1}^{S} R_sQ_s}{2RS} + \frac{\sum_{s=1}^{S} S_sQ_s}{2S^2} = 0.010,$$

where $P_s = (a_s + d_s)/T_s$ and $Q_s = (b_s + c_s)/T_s$. Recall that the cluster effect is a measure of the effective sample size. This means that we need about 1.95 more observations to maintain the same level of precision compared to a sample with no cluster effects.

Alternatively, instead of using the RBG formula which does assume a common odds ratio, we could simply use our adjustment but assume independence of the observations. We can ignore clusters and compute

$$v_{\text{Ind}} = \frac{T}{T-1}\left[\sum_{i=1}^{C}\sum_{s=1}^{S}\sum_{j=1}^{T_s}(z_{ijs} - \overline{z})^2\right] = 0.01002,$$

where $\overline{z} = \sum_{i=1}^{C}\sum_{s=1}^{S}\sum_{j=1}^{T_s} z_{ijs}/T$ and $T = 2343$ is the total number of observations. We obtain the following cluster effect:

$$\text{cluster effect} = \frac{\mathsf{Var}_{\text{adj}}(\ln(\widehat{\text{OR}}_{MH})}{v_{\text{Ind}}} = 1.942.$$

4 Concluding Remarks

We have presented a method to adjust the Mantel-Haenzsel test statistic and odds ratio for clustering. The method is robust in the sense that we make no assumption of a common odds ratio nor do we assume a constant intra-cluster correlation. It is a cluster adjustment for multinomially clustered data. That is, we assume that we have data from a random sample of clusters which could have multiple levels, and that the observations are then cross-classified according to the stratifier and outcome and exposure variables.

References

[1] Hauck, W.W. (1979). The large sample variance of the Mantel-Haenszel estimator of a common odds ratio. *Psychometrica.* **35,** 817-819.

[2] Mantel, N. and Haenszel, W. (1959). Statistical aspects of the analysis of data from retrospective studies of disease. *Journal of the National Cancer Institute* **22**, 719-748.

[3] Mosteller, F. (1968). Association and Estimation in Contingency Tables. *Journal of the American Statistical Association* **63,** 1-28.

[4] Williams, R.L. (2000) A Note on Robust Variance Estimation for Cluster-Correlated Data. *Biometrics*, textbf56 645-646.

[5] Weerasekera, D.R. and Bennett, S. (1992) Adjustement to the Mantel-Haenszel Test for Data from Sratified Multistage Surveys. *Statistics in Medicine* **11**, 603-616.

[6] Woolf, B. (1955). On estimating the relationship between blood group and disease. *Annals of Human Genetics.* **19,** 251-253.

[7] Miettinen, O.S. (1976) Estimability and estimation in case referent studies. *Am. J. Epidemiol.* **103**, 226235.

[8] Robins, J. and Breslow, N. and Greenland, S. (1986). Estimators of the Mantel-Haenszel variance consistent in both sparse data and large-strata limiting models. *Biometrics.* **42,** 311-323.
